# Incidence, risk factors, and prognosis of acute exacerbation of rheumatoid arthritis-associated interstitial lung disease: a systematic review and meta-analysis

**DOI:** 10.1186/s12890-023-02532-2

**Published:** 2023-07-11

**Authors:** Maosheng Xie, Chao Zhu, Yujin Ye

**Affiliations:** Department of Rheumatology and Immunology, The First Affiliated Hospital, Sun Yat-sen University, Guangzhou, China

**Keywords:** Rheumatoid arthritis, Interstitial lung disease, Acute exacerbation, Meta-analysis

## Abstract

**Introduction:**

Acute exacerbation (AE) is a devastating complication of rheumatoid arthritis-associated interstitial lung disease (RA-ILD) and leads to high mortality. This study aimed to investigate the incidence, risk factors, and prognosis of acute exacerbation of rheumatoid arthritis-associated interstitial lung disease (AE-RA-ILD).

**Methods:**

PubMed, EMBASE, Web of Science, and Medline were searched through 8 February 2023. Two independent researchers selected eligible articles and extracted available data. The Newcastle Ottawa Scale was used to assess the methodological quality of studies used for meta-analysis. The incidence and prognosis of AE-RA-ILD were investigated. Weighted mean differences (WMDs) with corresponding 95% confidence intervals (CIs) and pooled odds ratios (ORs) with 95% CIs were calculated to explore the risk factors of AE in RA-ILD.

**Results:**

Twenty-one of 1,589 articles were eligible. A total of 385 patients with AE-RA-ILD, of whom 53.5% were male, were included. The frequency of AE in patients with RA-ILD ranged from 6.3 to 55.6%. The 1-year and 5-year AE incidences were 2.6–11.1% and 11–29.4%, respectively. The all-cause mortality rate of AE-RA-ILD was 12.6–27.9% at 30 days and 16.7–48.3% at 90 days. Age at RA diagnosis (WMD: 3.61, 95% CI: 0.22–7.01), male sex (OR: 1.60, 95% CI:1.16–2.21), smoking (OR: 1.50, 95% CI: 1.08–2.08), lower forced vital capacity predicted (FVC%; WMD: −8.63, 95% CI: −14.68 to − 2.58), and definite usual interstitial pneumonia (UIP) pattern (OR: 1.92, 95% CI: 1.15–3.22) were the risk factors of AE-RA-ILD. Moreover, the use of corticosteroids, methotrexate, and biological disease-modifying anti-rheumatic drugs, was not associated with AE-RA-ILD.

**Conclusion:**

AE-RA-ILD was not rare and had a poor prognosis. Age at RA diagnosis, male sex, smoking, lower FVC%, and definite UIP pattern increased the risk of AE-RA-ILD. The use of medications, especially methotrexate and biological disease-modifying anti-rheumatic drugs, may not be related to AE-RA-ILD.

**Registration:**

CRD42023396772.

**Supplementary Information:**

The online version contains supplementary material available at 10.1186/s12890-023-02532-2.

## Background

Rheumatoid arthritis (RA) is an inflammatory joint disease that involves both genetic and environmental factors and can lead to joint damage and irreversible deformity [1]. Interstitial lung disease (ILD) is a major extra-articular manifestation of RA and increases mortality in patients with RA [2]. The median survival in rheumatoid arthritis-associated interstitial lung disease (RA-ILD) ranges from 3 to 10 years and approximately 35.9% of patients with RA-ILD die within 5 years of diagnosis [3–5]. RA-ILD and IPF share many clinical features and have similarities in genetic susceptibility [6]. As with IPF, RA-ILD with usual interstitial pneumonia (UIP) pattern is associated with lower survival [7]. Acute exacerbation (AE) is a life-threatening complication of RA-ILD with high mortality [8]. AE was first described in three patients with idiopathic pulmonary fibrosis (IPF) in 1993. These patients developed influenza-like symptoms with newly developing diffuse pulmonary infiltrates based on pre-existing chronic interstitial lung disease [9]. Patients with IPF can develop AE at any time, characterized by acute respiratory deterioration of unknown cause [10]. AE is not limited to IPF and can be present in a variety of ILDs, including rheumatic disease-associated ILD [11]. A previous meta-analysis summarized the incidence, risk factors, and prognosis of AE in systemic autoimmune disease-associated ILD [12]. The most predominant subtype of RA-ILD is the UIP pattern, whereas the nonspecific interstitial pneumonia pattern is more common in other rheumatic disease-associated ILD [13–16]. Considering the poor prognosis of acute exacerbation of rheumatoid arthritis-associated interstitial lung disease (AE-RA-ILD), we conducted a systematic review and meta-analysis to investigate the incidence, risk factors, and prognosis of AE-RA-ILD. The protocol of this study was registered with the International Prospective Register of Systematic Reviews (CRD42023396772).

## Methods

### Search strategy

We conducted this study in accordance with the Preferred Reporting Items for Systematic Reviews and Meta-Analyses guidelines [17]. Database retrieval and data extraction were performed independently by two researchers. Subject headings and text words such as “rheumatoid arthritis,” “interstitial lung disease,” and “exacerbation” were searched in electronic databases such as PubMed, Web of Science, Medline (Ovid), and EMBASE through 8 February 2023. The references of eligible articles and relevant reviews were also screened to identify additional studies.

### Eligibility criteria

Two researchers independently evaluated the eligibility of each study by screening the title and abstract and, if necessary, reading the full text. Studies were included if they indicated the incidence or risk factors of AE in RA-ILD patients, or the prognosis of AE-RA-ILD. The classification of RA was based on the 1987 or 2010 American College of Rheumatology/European League Against Rheumatism classification criteria for RA [18, 19]. ILD was diagnosed using radiology or biopsy and the pattern was classified following official guidelines [20–22]. AE was first proposed in IPF patients and then applied to RA-ILD with the following slight modifications: unexplained worsening or development of dyspnea within 30 days of RA-ILD, new bilateral ground-glass opacity and/or consolidation superimposed on a background pattern of ILD, and alternative causes such as heart failure were excluded [23]. Studies that did not meet the definition of AE-RA-ILD were excluded, such as those considered to have developed AE in RA-ILD patients who received high-dose methylprednisolone at the beginning of their hospitalization [24]. We only included articles published in English. If overlapping cohorts were observed, we chose the study with the larger sample size. Case reports, editorials, reviews, and conference abstracts were excluded.

### Data collection and risk of bias assessment

Two researchers extracted the first author’s name, year of publication, study location, study design, sample size and demographic features, disease incidence, potential risk factors, and prognosis. The Newcastle Ottawa scale (NOS), in which a score ≥ 7 indicated high-quality research, was used to assess the quality of articles. We resolved disagreements through discussion and reached consensus.

### Statistical analysis

All statistics were performed using Review Manager version 5.4 (Cochrane Collaboration, Oxford, UK). Continuous variables presented by median (range) or median (quartile) were converted to mean and standard deviation according to the reported formula [25]. To count data, we extracted the frequency of exposure factors in the exposure and control groups. Weighted mean differences (WMDs) with corresponding 95% confidence intervals (CIs) and pooled odds ratios (ORs) with 95% CIs were calculated using the Mantel–Haenszel fixed-effects or random-effects models when risk factors were presented in more than one study [26]. The fixed-effects model was used when no heterogeneity was observed between studies; otherwise, we chose the random-effects model. Heterogeneity across studies was expressed as I^2^, and a p-value < 0.05 was considered statistically significant [27]. Subgroup analysis was performed based on the definitions of UIP pattern in the studies. Forest plots were used to present the results of combined studies, and publication bias was not assessed because of the limited number of studies. The effect estimates of risk factors in multivariate models were qualitatively described.

## Results

### Study selection

**Fig. 1:**
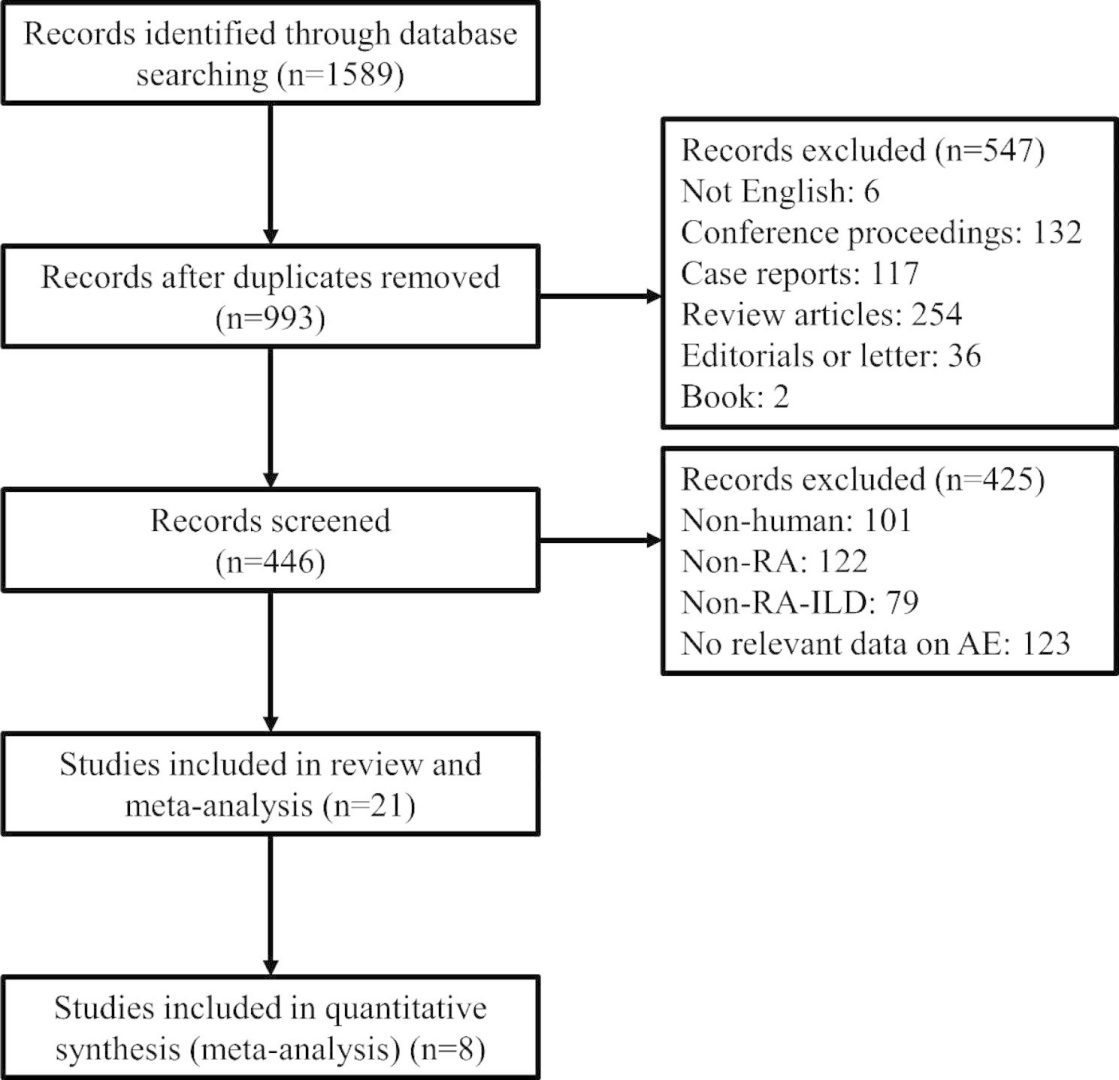
Study flow diagram. RA: Rheumatoid arthritis; RA-ILD: Rheumatoid arthritis-associated interstitial lung disease; AE: acute exacerbation

We collected 1,589 reports by searching PubMed, Web of Science, Medline (Ovid), and EMBASE. After removing 596 duplicates, we then excluded six reports not published in English, 132 conference proceedings, 117 case reports, 254 review articles, 36 editorials or letters, two books, and 425 irrelevant articles. The remaining 21 studies were included in the review, and eight studies were used for quantitative synthesis (Fig. 1). No eligible studies were identified by screening the references of eligible articles or related reviews. Six studies aimed to explore the risk factors of AE-RA-ILD in detail [28–33]. The remaining two studies addressed the relationship between the UIP pattern and AE-RA-ILD [34, 35].

### Study and subject characteristics

Among the 21 studies, Japan conducted the most studies (n = 13), followed by Korea (n = 3), China, India, Italy, Canada, and Saudi Arabia (n = 1 each). A total of 385 patients with AE-RA-ILD were enrolled. Approximately 53.5% of patients were male, and 56.6% had a history of smoking. The mean or median age at the onset of AE was 61.3–79.0 years. The frequency of AE in RA-ILD was reported in 16 studies and ranged from 6.3 to 55.6%. The 1-year AE incidence was 2.6–11.1% in four studies [30, 32, 36, 37], and the 5-year AE incidence was 11.0% and 29.4% in two studies [30, 32]. A total of 15 studies reported outcomes in patients with AE-RA-ILD, with an in-hospital all-cause mortality of 19.5% in one study [30], 30-day all-cause mortality of 12.6% and 27.9% in two studies [30, 31], and 90-day all-cause mortality of 16.7–48.3% in three studies (Table 1) [30, 38, 39]. The methodological quality of eight studies that were used for meta-analysis was assessed using NOS, and all studies were generally considered of medium-to-high quality.

**Table 1:** Characteristics of studies

Study	Country	Study design	Enrollment years	Numbers of AE-RA-ILD	Age (years) (at the onset of AE)	Male (n%)	Ever smoker (n%)	UIP pattern (n (%))	Follow-up lengths (months)	Frequency of AE-RA-ILD (n (%))/Incidence	Death of AE-RA-ILD (n(%))
Park [36]	Korea	Retrospective-cohort	1993–2006	3	mean 61.3	2 (66.7)	2/3 (66.7)	3 (100.0)^a^	-	3/18 (16.7)/11.1% (1-year)	3 (100.0) (overall)
Silva [65]	Canada	Case–control	1987–2006	3	mean 65.3	2 (66.7)	-	3 (100.0)^a^	-	-	1 (33.3) (overall)
Suda [37]	Japan	Retrospective- cohort	1987–2007	5	mean 64.4	4 (80.0)	5/5 (100.0)	2 (40.0)^a^	median 86^c^	5/25 (20.0)/2.6% (1-year)	4 (80.0) (overall)
Tachikawa [38]	Japan	Retrospective-cohort	2003–2009	6	mean 66.3	4 (66.7)	-	3 (50.0)^b^	-	-	1 (16.7) (90 days)
Song [66]	Korea	Retrospective-cohort	1991–2008	14	-	8 (57.1)	7/14 (50.0)	14 (100.0)^a and b^	-	14/84 (16.7)	13 (92.9) (overall)
**Hozumi** [32]	Japan	Retrospective-cohort	1995–2012	11	median 72	6 (54.5)	9/11 (81.8)	6 (54.5)^b^	median 102^c^	11/51 (21.6)/2.8% (1-year)	7 (63.6) (overall)
										11% (5-year)	
**Akiyama** [33]	Japan	Retrospective-cohort	2008–2014	6	-	2 (33.3)	2/5 (40.0)	3 (50.0)^b^	-	6/78 (7.7)	-
Toyoda [45]	Japan	Retrospective-cohort	2011–2015	6	-	-	-	-	-	-	1 (16.7) (overall)
Ota [67]	Japan	Retrospective-cohort	2006–2014	12	median 74.5	4 (33.3)	4/12 (33.3)	10 (83.3)^b^	median 19.5^d^	-	2 (16.7) (overall)
**Yamakawa** [34]	Japan	Retrospective-cohort	2012–2017	11	-	-	-	3 (27.3)^b^	-	11/96 (11.5)	-
Singh [68]	India	Retrospective-cohort	2013–2018	1	-	-	-	0 (0)^b^	-	1/16 (6.3)	0 (0) (overall)
Manfredi [11]	Italy	Prospective- cohort	2014–2015	2	-	0 (0)	0 (0)	1 (50.0)^b^	mean 9.5^c^	2/19 (10.5)	1 (50.0) (overall)
Wang [69]	China	Case–control	2016–2019	25	-	-	-	-	median 25.0^c^	25/45 (55.6)	-
**Suzuki** [35]	Japan	Retrospective-cohort	2008–2015	7	-	-	-	6 (85.7)^b^	-	7/71 (9.9)	-
Alhamad [70]	Saudi Arabia	Case–control study	2008–2019	15	-	-	-	-	-	15/43 (34.9)	-
**Tanaka** [28]	Japan	Retrospective-cohort	2010–2019	39	-	17 (43.6)	20/39 (51.3)	14 (36.8)^b^	median 36.0^c^	39/125 (31.2)	15 (38.5) (overall)
**Izuka** [31]	Japan	Retrospective-cohort	2007–2019	30	median 74	16 (53.3)	16/27 (59.3)	17 (56.7)^b^	-	30/165 (18.2)	13 (43.3) (overall)
											27.9% (30 days)
											37.8% (60 days)
Enomoto [48]	Japan	Retrospective-cohort	1999–2020	17	-	-	-	-	-	17/84 (20.2)	-
**Otsuka** [29]	Japan	Retrospective-cohort	2011–2019	27	median 79	14 (51.9)	14/27 (51.9)	23 (85.2)^b^	-	27/149 (18.1)	16 (59.3) (overall)
**Kwon** [30]	Korea	Retrospective-cohort	1997–2019	87	-	48 (55.2)	46/87 (52.9)	71 (81.6)^b^	median 47.7^c^	87/310 (28.1)/9.2% (1-year)	17 (19.5) (in hospital)
										19.8% (3-year)	12.6% (30 days)
										29.4% (5-year)	29.9% (90 days)
Hozumi [39]	Japan	Retrospective-cohort	2007–2019	58	median 73	35 (60.3)	39/58 (67.2)	44 (75.9)^b^	-	-	28 (48.3) (90 days)

Values presented as mean ± SD or number (percentage) unless otherwise specified. The eight studies used for the meta-analysis to explore the risk factors of AE-RA-ILD were bolded.

AE, acute exacerbation; RA, rheumatoid arthritis; ILD, interstitial lung disease; UIP, usual interstitial pneumonia.

^a^ Histologic pattern.

^b^ Radiological pattern.

^c^ Before the development of acute exacerbation.

^d^ after the development of acute exacerbation.

### Patient-specific variables

**Fig. 2:**
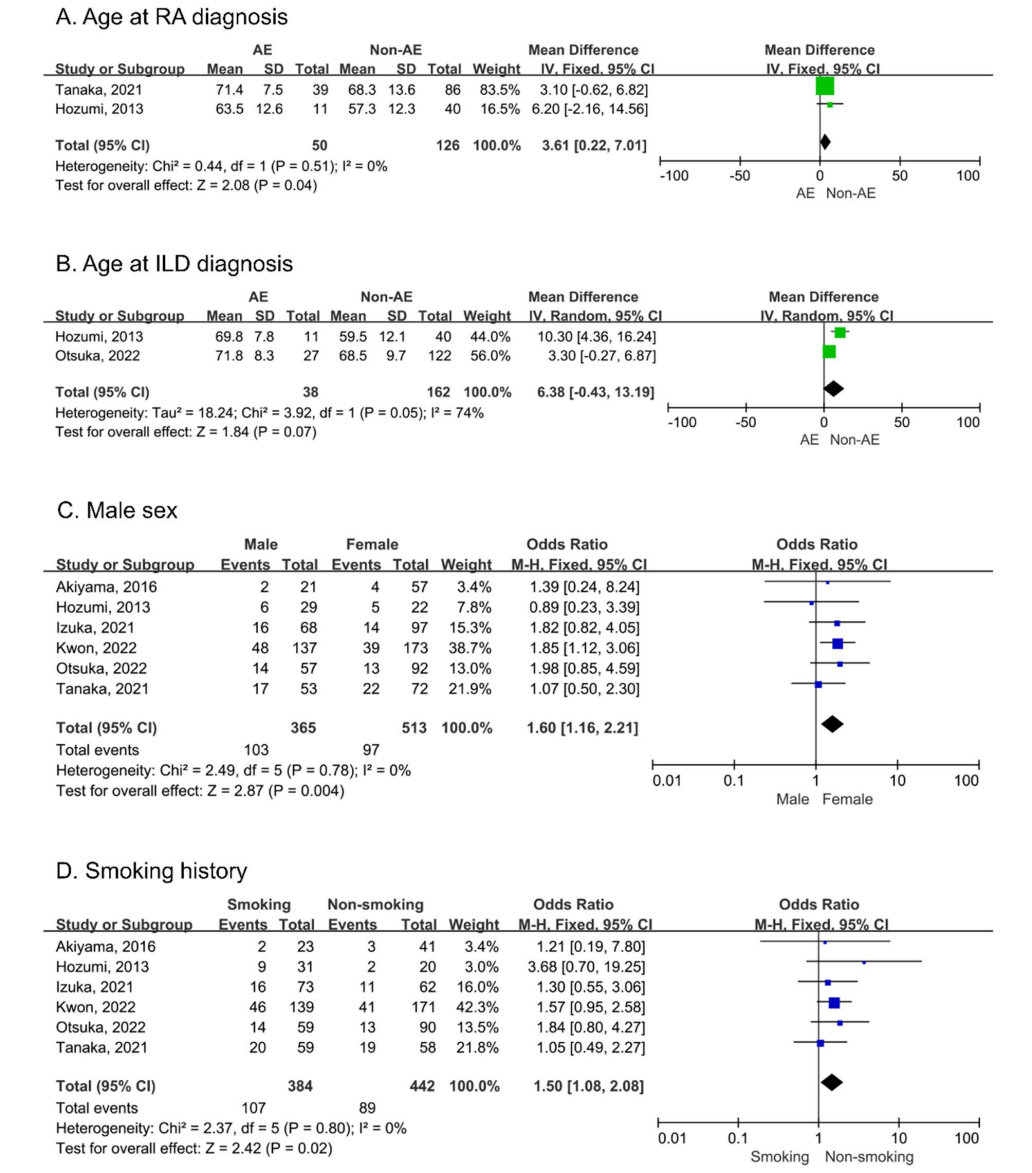
Forest plots for the correlation of age at RA diagnosis (A), age at ILD diagnosis (B), male sex (C), and smoking history (D) with AE in RA-ILD. RA: rheumatoid arthritis; ILD: interstitial lung disease; AE: acute exacerbation; RA-ILD: rheumatoid arthritis-associated interstitial lung disease

Six studies investigated the risk factors of AE in patients with RA-ILD. By combining the results of two studies, we observed that age at RA diagnosis was associated with AE-RA-ILD (WMD: 3.61, 95% CI: 0.22–7.01). No heterogeneity was observed between studies (I^2^ = 0%, p = 0.51; Fig. 2A) [28, 32]. No correlation was observed between age at ILD diagnosis and AE-RA-ILD (WMD: 6.38, 95% CI: −0.43–13.19), which was accompanied by heterogeneity (I^2^ = 74%, p = 0.05; Fig. 2B) [29, 32]. The pooled analysis suggested that the male sex was associated with an increased risk of AE-RA-ILD (OR: 1.60, 95% CI: 1.16–2.21), whereas no heterogeneity was observed between studies (I^2^ = 0%, p = 0.78; Fig. 2C) [28–33]. Smoking history included former and current smoking status. Our meta-analysis indicated an increased risk of AE in patients with RA-ILD with a smoking history (OR: 1.50, 95% CI: 1.08–2.08). No heterogeneity was found between studies (I^2^ = 0%, P = 0.80; Fig. 2D) [28–33]. Moreover, the multivariate analysis in one study showed that smoking was associated with AE-RA-ILD (HR: 1.762, p = 0.013) [30].

### RA-specific variables

In the six studies designed to investigate risk factors for AE-RA-ILD, no significant differences were observed between AE-RA-ILD and non-AE-RA-ILD groups, although rheumatoid factor (RF) and anti-cyclic citrulline polypeptide antibody (ACPA) were collected at different times and were expressed in different ways (i.e., positive or titer) [28–33]. We did not perform a meta-analysis because the criteria for positivity varied across studies. In patients with RA-ILD treated with tocilizumab, no significant difference in disease activity was observed between AE and non-AE groups at baseline. Univariate analysis suggested that a clinical disease activity index > 10 at 24 weeks was a risk factor for AE (OR: 4.7, 95% CI: 2.1–10.4) [33]. However, no significant differences in disease activity were observed between AE-RA-ILD and non-AE-RA-ILD groups at baseline, during the follow-up observation period, or at the onset of AE [28, 29, 32]. We did not perform a pooled analysis because disease activity was assessed at different times.

### ILD-specific variables

**Fig. 3:**
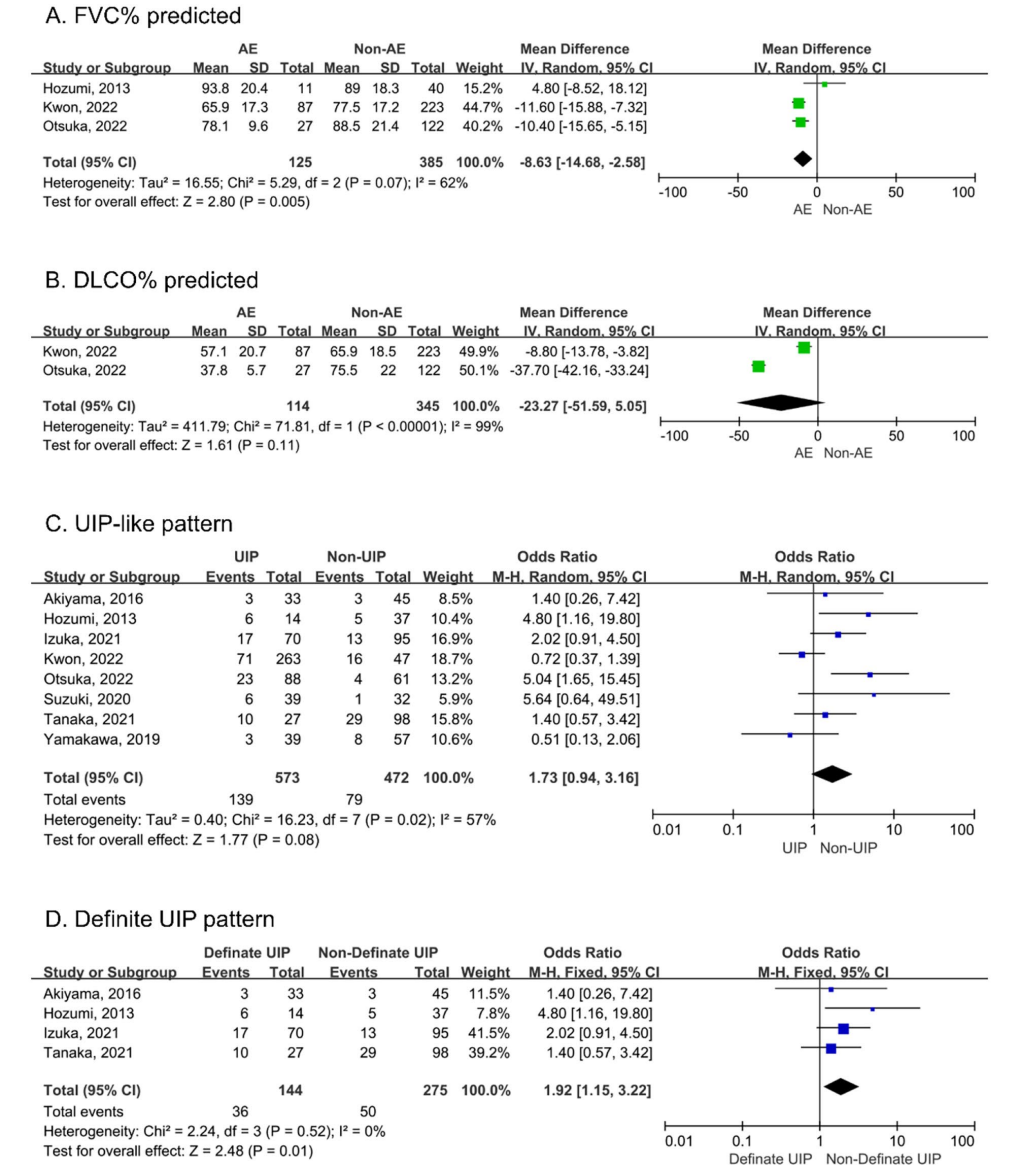
Forest plots for the correlation of FVC% (A), DLCO% (B), UIP-like pattern (C), and definite UIP pattern (D) with AE in RA-ILD. FVC%: percentage predicted forced vital capacity; DLCO%:  percentage predicted diffusing capacity of the lung for carbon monoxide; UIP: usual interstitial pneumonia

We found that percentage predicted forced vital capacity (FVC%) was associated with AE-RA-ILD (WMD: −8.63, 95% CI: −14.68 to − 2.58). Heterogeneity between studies was observed (I^2^ = 62%, P = 0.07; Fig. 3A) [29, 30, 32]. In contrast, percentage predicted diffusing capacity of the lung for carbon monoxide (DLCO%) was not related to the occurrence of AE (WMD: −23.27, 95% CI: −51.59 to 5.05), and high heterogeneity was observed (I^2^ = 99%, P < 0.00001; Fig. 3B) [29, 30]. Eight studies addressed the relationship between the UIP-like pattern on high-resolution computed tomography and AE-RA-ILD [28–35]. Our meta-analysis found that the UIP-like pattern was not associated with AE-RA-ILD (OR: 1.73, 95% CI: 0.94–3.16). Heterogeneity across studies was observed (I^2^ = 57%, P = 0.02; Fig. 3C). We performed a subgroup analysis because the UIP pattern in four of these studies was based on the definite UIP pattern described in the 2011 classification criteria [28, 31–33]. Other studies used different classification criteria and the UIP pattern contained possible UIP [29, 30, 34, 35]. The definite UIP pattern increased the risk of AE (OR: 1.92, 95% CI: 1.15–3.22), and heterogeneity was significantly reduced (I^2^ = 0%, P = 0.52; Fig. 3D). Two of the three studies showed that Krebs von den Lungen-6 (KL-6) was significantly higher in the group with AE-RA-ILD compared with the non-AE-RA-ILD group [28, 29, 33]. In the multivariate analysis, the annual variation rate of KL-6 was associated with AE-RA-ILD (HR: 3.37, 95% CI: 1.16–8.87) [28]. We did not perform a meta-analysis because of differing collection times across studies and the limited number of studies.

### Treatment upon AE occurrence or during last visit

**Fig. 4:**
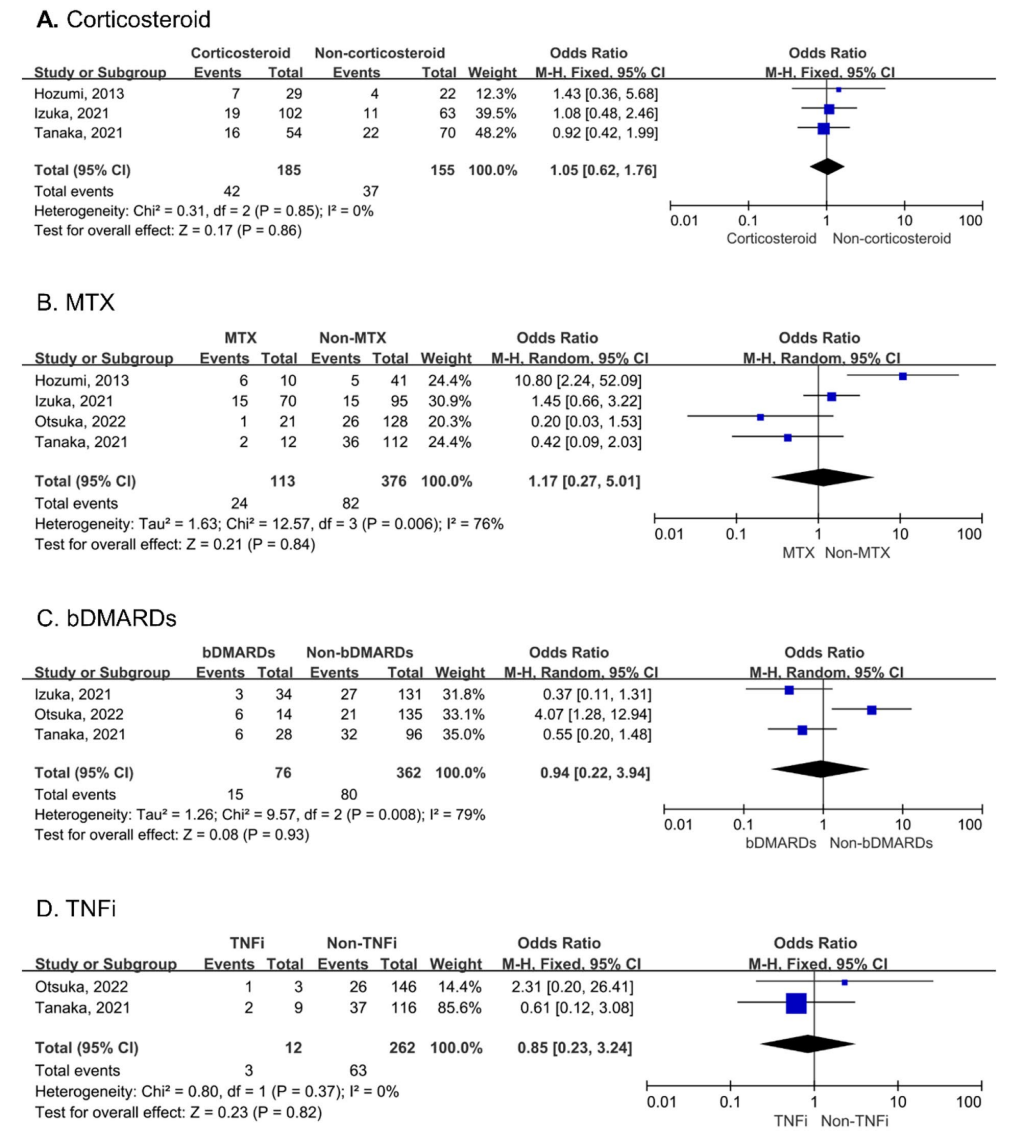
Forest plots for the correlation of corticosteroids (A), MTX (B), bDMARDs (C), and tumor necrosis factor inhibitors (D) with AE in RA-ILD. MTX: methotrexate; bDMARDs: biological disease-modifying anti-rheumatic drugs; AE: acute exacerbation; RA-ILD: rheumatoid arthritis-associated interstitial lung disease

The pooled results of three studies suggested that corticosteroids did not increase the risk of AE-RA-ILD (OR: 1.05, 95% CI: 0.62–1.76) [28, 31, 32]. No heterogeneity was observed (I^2^ = 0%, P = 0.85; Fig. 4A). Our meta-analysis showed that methotrexate (MTX) was not associated with AE (OR: 1.17, 95% CI: 0.27–5.01) [28, 29, 31, 32]. Heterogeneity across studies was high (I^2^ = 76%, P = 0.006; Fig. 4B). The pooled analysis showed that biological disease-modifying anti-rheumatic drugs (bDMARDs) were not associated with the occurrence of AE (OR: 0.94, 95% CI: 0.22–3.94) [28, 29, 31]. High heterogeneity was observed (I^2^ = 79%, P = 0.008; Fig. 4C). Furthermore, we found that tumor necrosis factor inhibitors were not associated with the occurrence of AE (OR: 0.85, 95% CI: 0.23–3.24) [28, 29]. No heterogeneity was observed (I^2^ = 0%, P = 0.37; Fig. 4D).

## Discussion

In this literature review and meta-analysis, we included 21 studies with a total of 385 patients with AE-RA-ILD. The mean or median age at the onset of AE was 61.3–79.0 years. The frequency of AE in patients with RA-ILD ranged from 6.3 to 55.6%, the 1-year AE incidence was 2.6–11.1%, and the 5-year AE incidence was between 11% and 29.4%. All-cause mortality was 12.6–27.9% at 30 days and 16.7–48.3% at 90 days. Age at RA diagnosis, male sex, smoking, lower FVC%, and definite UIP pattern on high-resolution computed tomography increased the risk of AE-RA-ILD. The use of medications such as corticosteroids, bDMARDs, and especially MTX was not associated with AE-RA-ILD.

The mean or median age at onset of AE-RA-ILD was 61.3–79.0 years, which was higher than that of AE-RD-ILD (45.8–74.5 years) [12]. The 1-year incidence of AE-RA-ILD was 2.6–11.1%, which was lower than that of AE in IPF patients (AE-IPF) (8.5–14.2%) [40, 41]. The 1-year incidences of AE-RD-ILD were 1.25% and 3.3% in two studies; these rates appeared lower than those for AE-RA-ILD and AE-IPF [36, 37]. The all-cause mortality rates of AE-RA-ILD and AE-IPF at 90 days were 16.7–48.3% [30, 38, 39] and 36.1–69.0% [38, 39, 42]. The all-cause mortality of AE-RD-ILD was 30–46.7% at 90 days [38, 43–45]. AE-IPF appeared to have the worst prognosis. Unexpectedly, the mortality rate of AE-RD-ILD at 90 days was not significantly lower than that of AE-RA-ILD; this could be attributable to the fact that AE-RD-ILD was dominated by RA and polymyositis/dermatomyositis (PM/DM) [12]. The prognosis of AE in PM/DM patients was poor: 25 patients (39.1%) died in hospital or within 2 weeks of hospital discharge [46]. Differing findings across AE-RD-ILD studies may be closely related to the types of underlying rheumatic disease.

Age at RA diagnosis, male sex, and smoking were associated with AE-RA-ILD in our pooled analysis. Moreover, older age, male sex, and smoking were associated with increased mortality of RA-ILD in a recent meta-analysis [47]. One study suggested that in patients with RA-ILD, the proportion of ILD diagnosis before RA onset was significantly higher in the AE group (33.3%) than in the non-AE group (11.5%) [29]. In a Korean cohort of patients with RA-ILD, ILD was diagnosed before RA in 22.9% of patients [30]. Age at diagnosis of RA rather than ILD may account for these differences. In a study of AE-RD-ILD with patients that predominantly had RA, most patients were older male smokers [48]. Male sex was also a risk factor for progressive RA-ILD [49]. Smoking not only increased the risk of RA but also was associated with lung involvement in RA [50]. However, smoking was not associated with AE-RD-ILD [12]. We analyzed the three studies included in this meta-analysis. Cao et al.’s study had the largest sample size, and primary Sjögren’s syndrome accounted for 35.4% of the 70 cases of AE-RD-ILD [51]. Unlike in RA, smoking was not a risk factor for ILD development and progression in primary Sjögren’s syndrome [52, 53]. These findings may explain differences in smoking as a risk factor.

Although RA-ILD was often accompanied by high titers of RF and ACPA, no studies have shown that RF and ACPA are associated with AE-RA-ILD [8]. In patients with RA-ILD treated with tocilizumab, poorly controlled disease activity was associated with AE [33]. However, the results of two studies suggested no significant difference between AE and non-AE groups, either at the onset of AE, at the last follow-up, or in the AE-free period [28, 32]. In a recent study in which patients with RA-ILD were followed annually for 3 years, no correlation was observed between lung function trajectory and disease activity [54]. Overall, the correlation between disease activity and AE-RA-ILD remains unclear.

Consistent with the result of a multivariate analysis [30], lower baseline FVC% indicated an increased risk of AE-RA-ILD in our meta-analysis. Lower FVC% was also a risk factor for AE in patients with IPF [26]. Our pooled analysis suggested that DLCO% was not associated with AE-RA-ILD. However, we noticed that both studies included in the analysis suggested that lower DLCO% was a risk factor for AE-RA-ILD and that considerable heterogeneity was observed between the two studies. Lower DLCO% was associated with an increased risk of AE-RA-ILD in a multivariate analysis [29]. Therefore, this result must be viewed with caution. More research on the relationship between lung function tests, especially DLCO%, and AE-RA-ILD are needed. Next, by pooling eight studies, we observed that the UIP pattern on high-resolution computed tomography was not associated with AE-RA-ILD. Heterogeneity was observed across the studies. We noticed that some studies included the definite UIP pattern, whereas other studies also included the possible UIP pattern. Our subgroup analysis found that the definite UIP pattern increased the risk of AE-RA-ILD; no heterogeneity was observed between studies. This finding explained why the UIP-like pattern was not associated with AE-RA-ILD in a large Korean cohort study [30]. In contrast, a multivariate analysis in which the definite UIP pattern described in the 2011 classification criteria was used suggested that the UIP pattern was a risk factor for AE-RA-ILD [31]. Given the major differences between definite and possible UIP patterns, our results suggested that honeycombing may be associated with AE.

Previous studies have shown that the use of MTX was associated with the development, progression, and acute exacerbation of RA-ILD [32, 55, 56]. A large prospective study suggested that MTX pneumonitis was rare [57]. Recent studies demonstrated that MTX improved lung function and prognosis in RA-ILD [58, 59]. However, the relationship between MTX use and AE-RA-ILD remains controversial. Our meta-analysis showed that the use of MTX did not increase the risk of AE-RA-ILD. Furthermore, the use of corticosteroids and bDMARDs, especially tumor necrosis factor inhibitors, was not associated with AE-RA-ILD. Whether bDMARDs can cause ILD and aggravate preexisting RA-ILD is a subject of controversy [60]. Our results partially alleviate concerns about medication use in patients with RA-ILD.

There are still some limitations. First, most of the included studies had been conducted in Asia, especially Japan, which was the same as the AE-IPF studies [61]. This may be due to the fact that the concept of AE was first proposed in Japan and thus received more attention [9]. The incidence of AE-IPF was similar in Asia and other ethnicities, further studies from other regions are needed to confirm the results of our study [62]. Second, some medications such as nintedanib have been shown to improve the prognosis of RA-ILD; however, nintedanib was not mentioned in the included studies [63, 64]. Third, given the limited number of studies and differing thresholds, some variables such as KL-6 and disease activity could not be pooled for analysis. Finally, given that few studies had focused on the prognostic factors of AE-RA-ILD, we did not conduct a meta-analysis.

## Conclusions

AE-RA-ILD was not rare and had a poor prognosis. Age at RA diagnosis, male sex, smoking, a lower FVC%, and a definite UIP pattern increased the risk of AE-RA-ILD. The use of medications, especially MTX and bDMARDs, was not associated with AE-RA-ILD.

## Electronic supplementary material

Below is the link to the electronic supplementary material.

Supplementary Material 1

Supplementary Material 2

Supplementary Material 3

## Data Availability

The dataset used and/or analysed during the current study will be available from the corresponding author on a reasonable request after the final result is published in a journal.

## Abbreviations

***AE***: Acute exacerbation
***ACPA***: Anti-cyclic citrullinated peptide antibody
***bDMARDs***: biological disease-modifying anti-rheumatic drugs
***CI***: Confidence interval
***DLCO***: Diffusing capacity of the lung for carbon monoxide
***FVC***: Forced vital capacity
***ILD***: Interstitial lung disease
***IPF***: Idiopathic pulmonary fibrosis
***KL-6***: Krebs von den Lungen-6
***MTX***: methotrexate
***NOS***: Newcastle Ottawa scale
***OR***: Odds ratio
***PM/DM***: Polymyositis/dermatomyositis
***RA***: Rheumatoid arthritis
RF: Rheumatoid factor
***RA-ILD***: Rheumatoid arthritis-associated interstitial lung disease
***RD-ILD***: Rheumatic diseases-associated interstitial lung disease
***UIP***: Usual interstitial pneumonia
***WMD***: Weighted mean differences
