# Supplementary material for: Incidence, risk factors, and prognosis of acute exacerbation of rheumatoid arthritis-associated interstitial lung disease: a systematic review and meta-analysis
Source: BMC Pulm Med. 2023 Jul 11;23:255. doi: 10.1186/s12890-023-02532-2 (PMC10337154; doi:10.1186/s12890-023-02532-2)
Supplement: Supplementary file 2 — Supplementary Material 2 [file 12890_2023_2532_MOESM2_ESM.docx]

**Title**

Incidence, risk factors, and prognosis of acute exacerbation of rheumatoid arthritis-associated interstitial lung disease: a systematic review and meta-analysis

**Authors**

Maosheng Xie^1^, Chao Zhu^1^ and Yujin Ye^1*^

*Correspondence:

yeyujin@mail.sysu.edu.cn

^1^Department of Rheumatology and Immunology, The First Affiliated Hospital, Sun Yat-sen University, Guangzhou, China

Telephone: +86 13600490868

**e-Appendix:** Search terms for each electronic database

PubMed：

Search: ("Arthritis, Rheumatoid"[Mesh]) OR (Rheumatoid Arthritis) AND (("Lung Diseases, Interstitial"[Mesh]) OR (Diffuse Parenchymal Lung Disease) OR (Interstitial Lung Diseases) OR (Diffuse Parenchymal Lung Diseases) OR (Interstitial Lung Disease) OR (Lung Disease, Interstitial)) OR (Pneumonia, Interstitial) OR (Interstitial Pneumonia) OR (Interstitial Pneumonias) OR (Pneumonias, Interstitial) OR (Pneumonitis, Interstitial) OR (Interstitial Pneumonitides) OR (Interstitial Pneumonitis) OR (Pneumonitides, Interstitial) AND ("Symptom Flare Up"[Mesh]) OR (Flare Up, Symptom) OR (Flare Ups, Symptom) OR (Symptom Flare Ups)) OR (Symptom Flaring Up) OR (Flaring Up, Symptom) OR (Acute Symptom Flare) OR (Acute Symptom Flares) OR (Symptom Flare, Acute) OR (Symptom Flareup) OR (Flareup, Symptom) OR (Flareups, Symptom) OR (Symptom Flareups) OR (Symptom Flare-up) OR (Flare-up, Symptom) OR (Flare-ups, Symptom) OR (Symptom Flare-ups) OR (Symptom Exacerbation) OR (Exacerbation, Symptom) OR (Symptom Exacerbations) OR (Symptom Increase) OR (Increase, Symptom) OR (Increase, Symptom) OR (Magnification, Symptom) OR (Symptom Magnifications)) OR (Symptom Worsening) OR (Worsening, Symptom) OR (Symptom Exaggeration)) OR (Exaggeration, Symptom) OR (Symptom Exaggerations) OR ("Disease Progression"[Mesh]) OR (Disease Progressions) OR (Progression, Disease) OR (Progressions, Disease)) OR (Disease Exacerbation)

Medline（Ovid）：

1 exp Arthritis, Rheumatoid/ 123193

2 rheumatoid arthritis.mp. 118210

3 exp Lung Diseases, Interstitial/ 82886

4 exp Pulmonary Fibrosis/ 27117

5 (interstitial adj3 lung adj3 disease?).mp. 20277

6 (pulmonary adj3 fibros$).mp. 36116

7 (interstitial adj3 pneumoni$).mp. 12738

8 alveolitis.mp. 6520

9 exp Disease Progression/ 204328

10 (acute adj3 exacerbation?).mp. 13401

11 (disease adj3 progression?).mp. 289592

12 (disease adj3 exacerbation?).mp. 5221

13 deterioration?.mp. 104009

14 1 or 2 162297

15 3 or 4 or 5 or 6 or 7 or 8 102815

16 9 or 10 or 11 or 12 or 13 418493

17 14 and 15 and 16 261

Web of Science：

1 "Arthritis, Rheumatoid (Topic) OR Rheumatoid Arthritis (Topic) " All Databases 336799

2 "(TS= (interstitial NEAR/3 lung NEAR/3 disease$)) OR TS= (interstitial NEAR/3 pneumonia$)) OR TS=(alveolitis)) OR TS= (pulmonary NEAR/3 fibros*) " All Databases 107639

3 "(TS= (acute NEAR/3 exacerbation$)) OR TS= (disease NEAR/3 progression$) OR TS= (disease NEAR/3 exacerbation$) OR TS=(deterioration$) "All Databases 851721

4 "#1 AND #2 AND #3 " All Databases 490

EMBASE：

#1 'rheumatoid arthritis'/exp

#2 'arthritis deformans':ti,ab,kw OR 'arthritis, rheumatoid':ti,ab,kw OR 'arthrosis deformans':ti,ab,kw OR 'beauvais disease':ti,ab,kw OR 'chronic articular rheumatism':ti,ab,kw OR 'chronic polyarthritis':ti,ab,kw OR 'chronic rheumatoid arthritis':ti,ab,kw OR 'disease, beauvais':ti,ab,kw OR 'infantile rheumatoid arthritis':ti,ab,kw OR 'inflammatory arthritis':ti,ab,kw OR 'polyarthritis, primary chronic':ti,ab,kw OR 'primary chronic polyarthritis':ti,ab,kw OR rheumarthritis:ti,ab,kw OR 'rheumatic arthritis':ti,ab,kw OR 'rheumatic polyarthritis':ti,ab,kw OR 'rheumatism, chronic articular':ti,ab,kw

#3 'interstitial lung disease'/exp

#4 'diffuse interstitial pneumopathy':ti,ab,kw OR 'diffuse parenchyma lung disease':ti,ab,kw OR 'diffuse parenchymal lung disease':ti,ab,kw OR 'diffuse parenchymal pulmonary disease':ti,ab,kw OR 'diffuse parenchymal pulmonary disorder':ti,ab,kw OR 'interstitial lung diseases':ti,ab,kw OR 'interstitial lung disorder':ti,ab,kw OR 'interstitial pneumopathy':ti,ab,kw OR 'interstitial pulmonary disease':ti,ab,kw OR 'interstitial pulmonary disorder':ti,ab,kw OR 'lung disease, interstitial':ti,ab,kw OR 'lung diseases, interstitial':ti,ab,kw OR 'pneumopathy, interstitial':ti,ab,kw

#5 'disease exacerbation'/exp

#6 'aggravation, disease': ti,ab,kw OR 'disease aggravation':ti,ab,kw OR 'disease flare':ti,ab,kw OR 'disease progression':ti,ab,kw OR 'exacerbation, disease':ti,ab,kw

#7 #1 OR #2

#8 #3 OR #4

#9 #5 OR #6

#10 #7 AND #8 AND #9
